# Chloride Intracellular Channel Protein 1 Expression and Angiogenic Profile of Liver Metastasis of Digestive Origin

**DOI:** 10.3390/cimb45020091

**Published:** 2023-02-06

**Authors:** Amalia Raluca Ceausu, Alexandru Ciolofan, Alexandru Blidisel, Andrei Alexandru Cosma, Pusa Nela Gaje, Octavian Cretu

**Affiliations:** 1 Angiogenesis Research Center, Department of Microscopic Morphology/Histology, “Victor Babes” University of Medicine and Pharmacy, Timisoara, Sq. Eftimie Murgu No. 2, 300041 Timisoara, Timis, Romania; 2 Department of Surgical Semiology, “Victor Babes” University of Medicine and Pharmacy, Sq. Eftimie Murgu No. 2, 300041 Timisoara, Timis, Romania

**Keywords:** chloride intracellular channel 1, liver metastases, histological growth pattern

## Abstract

Chloride intracellular channel 1 (CLIC1) is involved in cell migration and metastasis. The histological growth patterns of liver metastasis are as follows: desmoplastic (d-HGP), replacement (r-HGP), pushing (p-HGP), and mixed. The aim of this study was to evaluate the relation between HGP, angiogenesis, and CLIC1 expression. Materials and Methods: A total of 40 cases of primary tumors and their LM: d-HGP (12 cases), r-HGP (13 cases), and p-HGP (15 cases), were evaluated through simple and double immunostaining. CLIC1 assessment was conducted as follows: scores of 0 (less than 10% of positive cells), 1 (10–30%), 2 (30–50%), or 3 (more than 50%) were assigned. Heterogeneous CLIC1 expression was found. CLIC1 in primary tumors correlated with grade G for all cases of LM with a p-HGP (*p* = 0.004). The CLIC1 score for LMs with an r-HGP correlated with grade G of the corresponding primary tumor (*p* = 0.027). CLIC1 and CD34+/Ki67+ vessels (*p* = 0.006) correlated in primary tumors. CLIC1 in primary tumors correlated with CD34+/Ki67+ vessels of LMs with a d HGP (*p* = 0.024). Conclusions: The CLIC1 score may have prognostic value, mainly for LMs with a p-HGP and r-HGP, and therapeutic value for LMs with a d-HGP.

## 1. Introduction

Colorectal cancer remains one of the most common malignancies in humans, with increased morbidity and specific mortality. Additionally, more than half of patients may develop liver metastasis, which is the leading cause of death [1]. Despite significant advances in diagnostic and therapeutic techniques, the survival rate of patients with liver metastasis remains low. Gastric tumors, which occupy fourth place in terms of frequency and second place in terms of morbidity, have shown liver metastasis at the time of diagnosis in more than a quarter of patients [2]. For pancreatic neoplasms, incidence and mortality are approximately equal. Likewise, the liver is one of the preferred sites of metastasis. However, unlike colorectal neoplasms, current guidelines do not recommend re-section of liver metastases for pancreatic neoplasms because no significant improvement in survival has been noticed [3].

Since 2017, when the first studies and agreements on the importance of histological growth patterns of liver metastases were established, numerous studies have supported their clinical importance for patients with liver metastases of different tumor types. These studies detailed the characteristics of histological growth patterns, their biology, and the main molecular and cellular mechanisms. Several types of liver metastases have been presented: desmoplastic, characterized by the existence of a band of connective tissue at the level of the invasion area; pushing type, in which the liver tissue is compressed by the metastasis; replacement, in which tumor cells penetrate between cords of hepatocytes, progressively replacing them; and the mixed model, in which two of the previous models are found [4,5].

In an attempt to solve the previously mentioned problems, numerous markers have been studied for primary tumor and liver metastasis with a digestive origin: CXCL12, CXCR4, TP53, APC, KRAS, PIK3CA, SMAD4, TCF7L2, BRAF, SOX9, NOTCH3, PTPRT, CTNNB1, ATM, EMX-2, TGF beta, SDF-1, Galectin-3, Ki67, MMP 7, beta catenin, MET-tyrosine kinase receptor, HER2, CA 19-9, AKT1, CDKN2A, ERBB2, IL6, STAT3, CASP3, NOTCH1, and CTNNB1 [6,7,8].

p64 was the first member to be identified, followed by p64H1, which were later renamed CLIC1 and CLIC4, respectively. Chloride intracellular channel 1 (CLIC1) is the most studied member of the family. This enzyme has been identified in the nucleus, cytoplasm, and cell membrane; therefore, it may play an important role in normal physiological cell states, such as the proliferation and differentiation of cells, changes in cell volume, and regulation of membrane potential. It has also been identified in pathological conditions, being involved in cell migration, especially in invasion and metastasis [9]. Regarding CLIC4 involvement in neoplasia, it has been shown to be overexpressed in malignant pleural mesothelioma [10]. These data underline the importance of CLICs in cancer.

Chloride intracellular channel 1 (CLIC1) has been shown to be overexpressed in many cancers (hepatocellular carcinoma, lung cancer, gall bladder cancer, pancreatic ductal adenocarcinoma, glioma, renal cell carcinoma, and gastric cancer) and associated with progression and metastasis; however, the mechanisms and pathways of action have not been fully elucidated [11,12]. One of the proposed pathways is the activation of PIP5K (phosphatidylinositol-4-phosphate 5-kinase). At the cellular level, these enzymes are responsible for regulating various cellular processes such as G protein-coupled receptor (GPCR) signaling, vesicular transport, chemotaxis, and cell movement. Another important role of CLIC1 that has already been demonstrated is its involvement in the growth, migration, proliferation, and angiogenic processes. CLIC1 began to be considered a potential therapeutic target when data from the literature demonstrated its involvement in the remodeling of actin filaments for the development of lamellipodia and invadopodia, useful for cell movements, their capacity for invasion, and the formation of metastases [13,14]. CLICs are proteins, which have several unexplored functions that may be very important to cancer, such as their ion channel activity [15].

Based on these considerations, the aim of the present study was to assess the interrelations between histological growth patterns of liver metastases, CLIC1 immuno-expression, and angiogenesis in primary tumors and metastatic areas.

## 2. Materials and Methods

### 2.1. Patients and Biopsies

The present study included 41 cases of primary tumors and their corresponding liver metastases. The distribution was as follows: colorectal (30 cases), pancreatic (eight cases), and gastric (three cases). Signed consents forms were obtained from each patient, the principles of the Declaration of Helsinki were respected and the study was approved by the Ethics Committee of “Victor Babes” University of Medicine and Pharmacy Timisoara (no. 7339/22 April 2016).

### 2.2. Immunohistochemistry

The immunohistochemical method was performed with a Leica Bond Max Autostainer (Breckland, Linford Wood, UK). After the epitope retrieval solution (ER 2) was applied for 20 min, the peroxide blocking was realized by using hydrogen peroxide 3% for 5 min. The primary antibodies used were: CLIC1 (356.1, dilution 1:2000, incubation time—1 h at room temperature; from Santa Cruz Biotechnology, SC 81873, Santa Cruz, CA, USA); CD34 (clone QBEnd10, RTU, incubation time—20 min—Leica Biosystem Newcastle Ltd., Newcastle Upon Tyne, UK). The visualization was realized by using the Bond Polymer Refine Detection System (16 min). 3,3 Diaminobenzidine was used as the chromogen (10 min) and hematoxylin as the counterstain (10 min).

Double immunostaining. Ki67 was used as the primary antibody (FLEX Monoclonal Mouse Anti-Human Ki-67 Antigen, clone MIB-1, RTU, incubation for 20 min—Dako, Glostrup, Denmark). Incubation with the secondary antibody CD34 was carried out for 20 min, followed by visualization (Bond Polymer Refine Red Detection System—16 min), chromogen application and counterstaining. The entire immuno-histochemical procedure was performed with a Bond Max Autostainer (Leica Biosystem).

Microscopic evaluation and data analysis. The evaluation of the slides, pictures capture, and processing were performed using a Grundium Ocus 40 microscope (Hermiankatu 6G, 33720 Tampere, Finland) and Panoramic Viewer Pathology Software (3D Histech, Budapest, Hungary). The assessment of CLIC1-positive cells corresponds to the following scores: 0 (less than 10% of positive cells), 1 (10–30%), 2 (30–50%) and 3 (more than 50% of positive cells). The evaluation of the microvascular density was conducted according to Weidner et al. [16]. The statistical evaluation was conducted using IBM SPSS Statistics 28, and a *p*-value of < 0.05 was considered significant.

### 2.3. Bioinformatic Analysis

The Cancer Genome Cancers 2022 and Atlas (TCGA) databases were accessed by using the cBioPortal website (http://www.cbioportal.org, accessed on 26 January 2023) to find details related to the role and expression of CLIC1 in gastric, colorectal and pancreatic adenocarcinoma. By use of the GraphPad Prism 9.5.0, Graphpad Software, Inc., 2236 Avenida de la Playa La Jolla, San Diego, CA, USA the patients who expressed CLIC1 and liver metastases were divided into three groups according to histological growth factor (group A—desmoplastic growth pattern; group B—pushing-growth pattern and C—replacement histological growth pattern) and the survival analysis was made.

## 3. Results

Microscopic evaluation of HE-stained slides indicated the following distribution of primary tumors: well-differentiated (colorectal adenocarcinomas, 11 cases), moderately differentiated (colorectal adenocarcinoma, 10 cases; pancreatic ductal adenocarcinoma, three cases; and gastric tubular adenocarcinoma, two cases) and poorly differentiated (colorectal adenocarcinoma, nine cases; pancreatic ductal adenocarcinoma, four cases; and gastric adenocarcinoma intestinal type, one case). The histological growth patterns (HGPs) of their corresponding liver metastasis (LM), noticed on morphological staining, were as follows: desmoplastic (12 cases of colorectal origin), replacement (six cases of colorectal origin and seven cases of pancreatic origin), and pushing (12 cases of colorectal origin and three cases of gastric origin).

Primary colorectal tumors metastasized to the liver and that developed a desmoplastic growth pattern of LM (d HGP) were distributed as follows: well-differentiated (five cases), moderately differentiated (four cases), and poorly differentiated (three cases).

The vascular morphology of the primary tumor areas on CD34 immunostaining indicated the presence of vessels with a large, irregular lumen, vessels without a lumen, and vessels with pillar formation within the vessel lumen in the well-differentiated cases. A similar vessel morphology was found in the moderately and poorly differentiated cases.

The microscopic assessment revealed the following average values of microvascular density in the primary tumors (CD34+/Ki67− blood vessels): 11 (for well-differentiated primary tumors), 12.25 (moderately differentiated) and 26.33 (poorly differentiated). The average values of CD34+/Ki67+ blood vessels were: 18.60 (well-differentiated), 21.75 (moderately differentiated), and 26 (poorly differentiated).

The previous primary tumor developed LM with a d-HGP. At this level, CD34+/Ki67 negative vessels average values were: 9.6 (well-differentiated), 13.25 (moderately differentiated), and 17.33 (poorly differentiated). The CD34+/Ki67+ positive blood vessels’ average values were lower in the LM compared to the primary tumor: 0.4 (for well-differentiated), 0.75 (moderately differentiated), and 1 (poorly differentiated). The corresponding LM of the moderately differentiated colon adenocarcinoma, with a d-HGP, showed numerous wide lumen vessels in the desmoplastic area and fewer vessels, predominantly without a lumen, within the metastatic area. In the desmoplastic stroma of LM originating in the poorly differentiated colon adenocarcinoma, numerous vessels with intussusception aspects were noted.

All the LMs of gastric adenocarcinoma (three cases) included in the present study had a pushing HGP (p-HGP). Twelve of the colorectal adenocarcinomas developed metastasis with a p-HGP. The distribution of the average values of CD34+/Ki67− and CD34+/Ki67+ vessels in primary tumors and the corresponding LMs with a p-HGP according to the degree of differentiation is detailed in the table below (Table 1).

**Figure 1:**
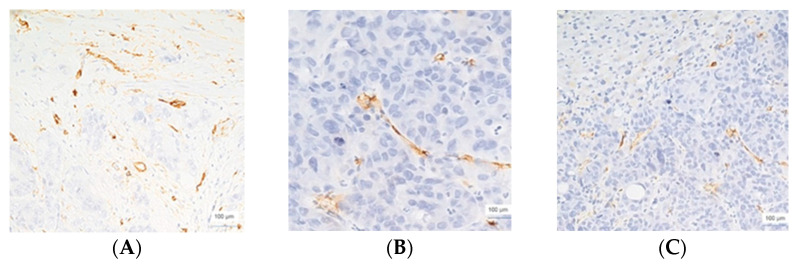
CD 34 immunoexpression, poorly differentiated colon adenocarcinomas, intratumoral vessels, ×200 magnification (**A**,**B**), and corresponding metastasis (p-HGP), ×200 magnification (**C**).

The blood vessel morphology of poorly differentiated colon and gastric adenocarcinomas and their corresponding LMs with a p-HGP showed vessels with a lumen as well as numerous narrow vessels or without a lumen (Figure 1). Changes in the portal space architecture were additionally noted.

All seven cases of pancreatic adenocarcinomas evaluated in the present study developed a replacement HGP (r-HGP) of LM. The average values of non-proliferative vessels in the primary tumors were 67 (moderately differentiated) and 90 (poorly differentiated). The proliferative vessel values were 0 (moderately differentiated) and 1 (poorly differentiated). Concerning LMs with an r-HGP, the identified average values were: 77 and 47.75 for non-proliferative vessels, and 0.66 and 0.25 for proliferative vessels.

**Figure 2:**
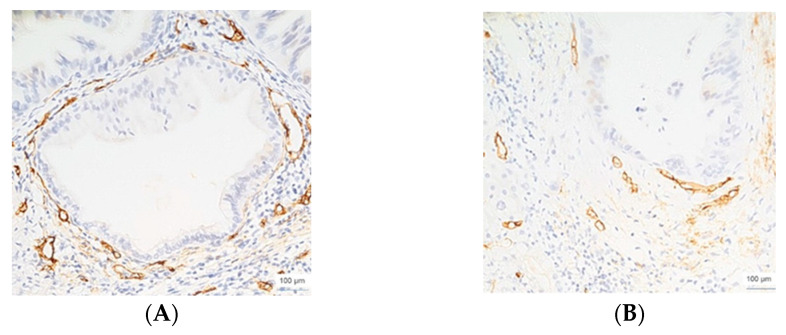
CD34 immunoexpression in moderately differentiated colon adenocarcinoma, ×200 magnification (**A**), and corresponding liver metastasis, replacement HGP, ×100 magnification **(B**).

From the colorectal adenocarcinomas, six cases showed an r-HGP (Figure 2). The average values of non-proliferative (CD34+/Ki67−) vessels in the primary tumor of colorectal adenocarcinoma were: 4 (well-differentiated), 20 (moderately differentiated), and 12 (poorly differentiated). Except for one case (poorly differentiated, with one proliferative vessel), all of the other cases did not present CD34+/Ki67+ proliferative endothelial cells. Their corresponding LM was characterized, in order, by the following average values of non-proliferative vessels: 3.66 (well-differentiated tumors), 15 (moderately differentiated tumors), and 31 (poorly differentiated tumors). Except for one case, all LM with an r-HGP did not present proliferative CD34+/Ki67+ vessels.

**Figure 3:**
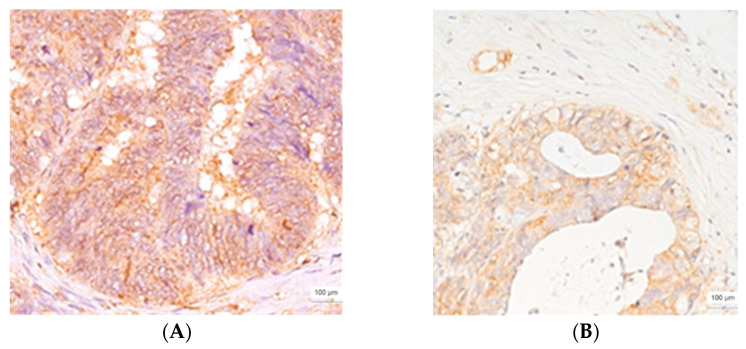
CLIC1 immunoexpression, colorectal adenocarcinoma, score of 2, ×400 magnification (**A**), and LM, desmoplastic HGP, score of 2, ×200 magnification (**B**).

All colorectal adenocarcinomas that developed LM with a d-HGP, had a value 2 for the score (Figure 3), except for one case, assessed with a score of 3. CLIC1 expression was heterogeneous in intensity, distribution, and pattern. Thus, cytoplasmic, cytoplasmic/membranous, nuclear and nuclear/cytoplasmic expression were noticed. Most of the LM with a d-HGP (66.66%) had a value of 3 for the CLIC1 score (eight cases). Four cases were quantified with a score of 2. The same expression pattern was found in LM. The vessels’ endothelium of the connective ring, from the periphery of the metastatic area, also expressed CLIC1.

**Figure 4:**
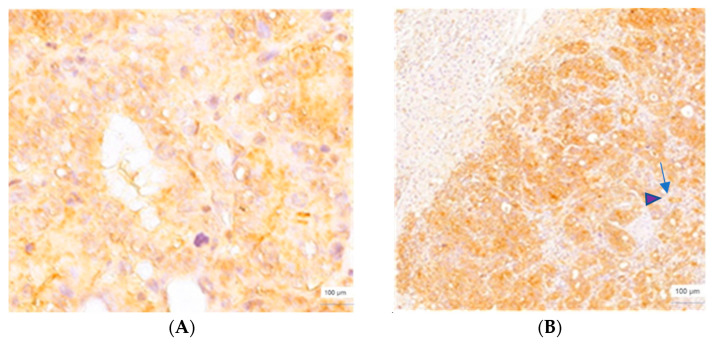
CLIC1 immunoexpression, gastric adenocarcinoma, score of 2, ×400 magnification (**A**), and corresponding LM, pushing HGP, score of 3, ×200 magnification (**B**). CLIC1 expression intensity in the cytoplasm (purple arrow 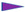) increased in the nucleus (blue arrow 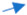) of the cell.

Evaluation of the colorectal and gastric adenocarcinomas revealed a value of 3 for the CLIC1 score (seven colorectal cases and one gastric case) and a value of 2 (five colorectal cases and two gastric cases). The following CLIC1 scores characterized their corresponding LMs with a p-HGP: 3 (five colorectal cases and two gastric cases) and 2 (seven colorectal cases and one gastric case). Gastric LMs with a p-HGP quantified with a score of 3 presented the highest intensity of CLIC1 expression at the periphery of the metastatic area (Figure 4). The nuclear expression pattern was noticed mainly at this level. CLIC1-positive sinusoids and cells with a dendritic morphology were noticed in the vicinity of the metastatic area. For the LMs with a p-HGP with a colorectal origin, the nuclear pattern and highest intensity of CLIC1 localization were maintained. A particular aspect was the presence of CLIC1-positive cells with a star-shape morphology, in the subcapsular area.

**Figure 5:**
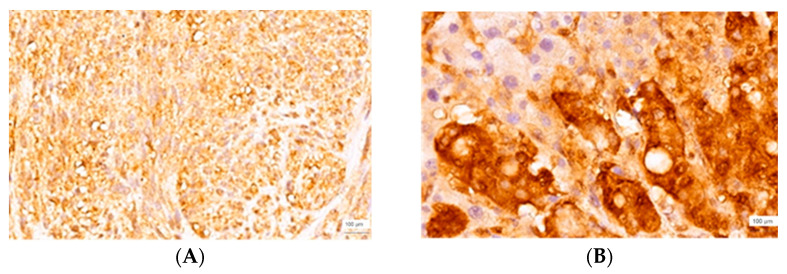
CLIC1 immunoexpression, pancreatic adenocarcinoma, score of 3, ×200 magnification (**A**) and corresponding LM, replacement HGP, score of 3, ×400 magnification (**B**).

Thirteen cases—six of colorectal origin and seven of pancreatic origin—developed LM with an r-HGP. A CLIC1 score of 3 was found in 50% of the colorectal adenocarcinomas, with a score of 2 being found for the other half. The distribution of the CLIC1 scores in LMs with an r-HGP of colorectal origin was: 3 (three cases), 2 (two cases), and 1 (one case). The CLIC1 scores of pancreatic adenocarcinomas were heterogeneous: 3 (three cases- Figure 5A), 2 (two cases), 1 (one case), and 0 (one case). Compared to the primary tumors, the majority of LMs with an r-HGP of pancreatic origin had a value of 3 for the score. Only one case had a value of 2 for the score.

CLIC1 nuclear expression was especially associated with the areas where the intensity of the reaction had a value of 3 (Figure 5B). The cells with a dendritic morphology were localized to the vicinity of the metastatic area and at the subcapsular level where networks were formed. The areas with cells with a star-shape morphology were accompanied by CLIC1-positivity in the sinusoids.

The CLIC1 score in the primary tumors was significantly correlated with the primary tumors’ proliferative vessels’ microvascular density (*p* = 0.006) and with the proliferative vessels of LMs with a d-HGP (*p* = 0.024).

The CLIC1 score for primary tumors was significantly correlated with grade G for all cases that developed LM with a p-HGP (*p* = 0.004). A significant correlation between the proliferative vessels from primary tumors and metastatic proliferative vessel density was noticed for all the cases that developed a p-HGP (*p* = 0.002).

The CLIC1 score for LMs with an r-HGP correlated with grade G of the corresponding primary tumors (*p* = 0.027). The only significant correlation was found between non-proliferative metastatic vessels’ density and the CLIC1 score for colorectal cases that developed LM with an r-HGP (*p* = 0.045). A similar correlation was noticed in colorectal primary tumors (*p* = 0.028).

**Figure 6:**
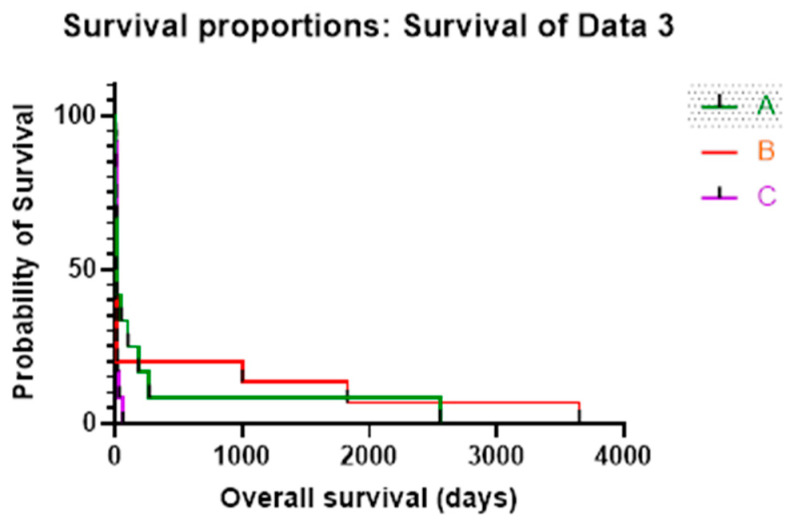
The overall survival in CLIC1 positive cases of LM, divided in three groups (A = d-HGP; B = p-HGP and C = r-HGP).

The overall survival rate of patients with liver metastases and CLIC 1 positivity was shown in the following figure (Figure 6).

The CLIC1 intensity of immunoexpression has a value of 3 in some endothelial cells. A heterogeneous expression of CLIC1 was noticed at the endothelial level. Related to the growth patterns of liver metastases, the desmoplastic type presented CLIC1-positive vessels predominantly in the connective ring at the periphery of the metastatic area. For the pushing and replacement types, the positivity of the vessels of the portal space and sinusoids was constantly found, the last especially in the p-HGP.

**Figure 7:**
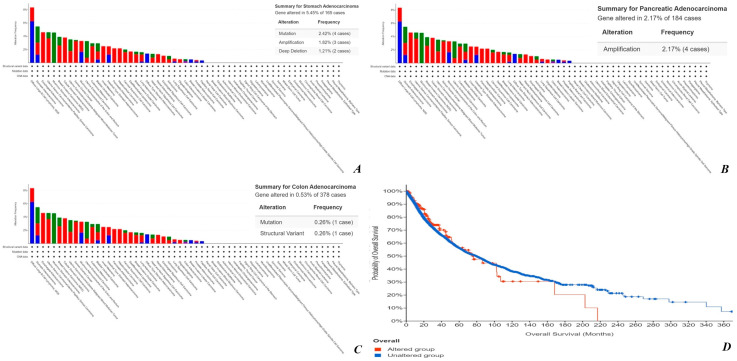
CLIC1 alteration from TCGA database in gastric adenocarcinoma (**A**), pancreatic adenocarcinoma (**B**) and colorectal adenocarcinoma (**C**). CLIC1 expression and overall survival interrelation (**D**).

A total number of 440 cases of stomach adenocarcinoma, 184 cases of pancreatic adenocarcinoma and 594 cases of colorectal adenocarcinoma may be identified from the TCGA databases. The CLIC1 alteration was found as following: gastric adenocarcinoma cases (3.86%), pancreatic adenocarcinoma cases (2.17%) and colorectal adenocarcinoma cases (1.01%). All these aspects may be noticed in Figure 7A–C. In our study, except one case of pancreatic adenocarcinoma, all of the primary tumors were CLIC1-positive. No results were found in TCGA databases regarding CLIC1 alteration in liver metastases of colorectal, pancreatic and gastric adenocarcinoma.

From the total number of cases—different tumor types (10.967 samples/10.953 patients)—159 were characterized by CLIC1 alteration. It may be noticed that high CLIC1 expression significantly reduces the overall survival (month) according to the Kaplan–Meier curve (Figure 7D).

## 4. Discussion

The HGP is considered as one of three independent predictors for overall survival in CRC. An association between the primary CRC histopathology and the HGP of the corresponding liver metastasis has been demonstrated [17]. In an attempt to reduce this influence, a recent study of over 4000 patients that analyzed the survival rate after CRLM surgery indicated the prognostic role of the HGP, this being equaled only by KRAS and BRAF mutational status [18]. Our study, which included 40 cases of primary tumors and corresponding LMs of digestive origin, can also be placed under having a statistical data influence; however, it may represent an additional signal of the usefulness of this parameter, alone or in combination, in prognostic, diagnostic, and therapeutic decisions.

Several angiogenic models have been proposed for LM of colorectal origin. Thus, for the LMs with an r-HGP, vascular co-option has been proposed by involving sinusoids that are co-opted by tumor cells. Hepatic metastases with a colorectal origin and p-HGP ensure their blood and nutrient supply through sprouting angiogenesis. The angiogenic model proposed for liver metastases with a colorectal origin and d-HGP is the sprouting type [19]. On the other hand, a study performed using an experimental model on the chick chorioallantoic membrane of an embryonated egg indicated metastasis with a d-HGP, cord-like structures formed by CD34+/Ki67+ cells, vessels with a wide lumen and proliferative endothelium, and vessels with large dimensions. Intraluminal pillars and the intussusception mechanism were found [20]. In the present study, sprouting and intussusception mechanisms were identified mainly in LMs with a d-HGP and p-HGP. Proliferative and non-proliferative vessels’ microvascular density in primary tumors was significantly correlated with metastatic proliferative vessels of LMs with a p-HGP.

There are six members in the CLIC family (CLIC1–6) [21], and apart from the widely reported channel activity, these proteins also play roles as enzymes, being present in their soluble form in the cytoplasm [22]. They interact with several cytoskeletal filaments and intracellular proteins. CLICs are extensively studied in cancer and tumor growth and display differential expression and localization in cancer cells during metastasis [23].

Knowing that colorectal cancer is one of the most common malignancies, it is vital for patients to undergo early detection and treatment. Several studies have demonstrated the expression of CLIC1 either in gastric cancer tissues or colon cancer cells; therefore, it is likely to be beneficial in clinical practice as a potential tumor marker [24]. According to our data, this is the first study to demonstrate CLIC1 expression in liver metastases. The present study showed the expression of CLIC1 with a heterogeneous distribution pattern, with intensity values between 1 and 3.

In normal conditions, the CLIC1 immunoexpression pattern was cytoplasmic. Some data showed heterogeneous expression patterns in renal cell carcinoma such as nuclear, nuclear/cytoplasmic, membranous, cytoplasmic/-membranous, nuclear/membranous, and nuclear/cytoplasmic/-membranous [25]. The membrane integration of CLIC1 accompanied by a higher tumor aggressiveness was underlined by Setti [26] in glioblastoma cases, as well as Peretti [27]. In the present study, the nuclear and cytoplasmic expressions of CLIC1 were associated with the highest intensity reaction and localization to the periphery of the metastatic area, mainly in liver metastases with a p-HGP and r-HGP.

Thuringer et al. [28] reported interactions between glioblastoma cells and endothelial cells, mediated by CLIC1. Tumor cells released it through extracellular vesicles. Endothelial cells captured it and developed sprout and tube formation in 3D matrices. In the present study, a correlation between CLIC1 expression and non-proliferative vessels from LMs with an r-HGP and proliferative vessels from LMs with a d-HGP was noted. The CLIC1 immunoexpression in the sinusoids and in the cells with a star-shape morphology, which especially localized to the vicinity of the metastatic area for the LMs with replacement and pushing HGPs and the subcapsular area for LMs with a d-HGP, may indicate the existence of different mechanisms of CLIC1 action, depending on the HGP of LM; however, more studies are needed.

CLIC1 has been reported as a potential biomarker and therapeutic target for some tumors such as oral squamous cell carcinoma, ovarian cancer, and hepatocellular carcinoma [29,30]. In hepatocellular carcinoma, CLIC1 expression has been correlated with angiogenesis by regulating VEGF-A [31]. The present study showed correlations between the CLIC1, the microvascular density of proliferative and non-proliferative vessels in the primary tumors, and LM, according to the HGP.

CLIC1 has been noticed to be overexpressed in pancreatic neoplasms. In addition, siRNA targeting CLIC1 mRNA with a subsequent decrease in CLIC1 expression leads to a decrease in cell proliferation [32]. This study demonstrated that CLIC1 expression is integral to the development and progression of pancreatic cancer. Jia et al. [33] showed an increased expression of CLIC1 in pancreatic ductal adenocarcinoma correlating a higher expression with a higher histological grade and increased tumor size. Few data are available in the literature regarding CLIC1 immunoexpression in liver metastases of pancreatic origin. In our study, CLIC1 was expressed with the highest score of 3 in all the LM cases, except one. In gastric cancer, the involvement of CLIC1 in metastatic progression was observed by reducing AMOT-p130 expression [34]. Scores of 2 and 3 in LM were found in the present study.

Furthermore, current treatment regimens do not affect colorectal r HGP lesions, and although they kill most cancer cells of d HGP lesions, there are some cells that survive within or adjacent to the desmoplastic ring that could give rise to new lesions [35].

Huang et al. [36] and Gururaja et al. [37] noticed an association between CLIC1 and CLIC 3 immunoexpression and an advanced state of HCC and survival in several different cancers. In the present study, the highest survival rate was found in patients with the desmoplastic and pushing type of LM, with CLIC1 scores of 3 and Avastin treatment.

## 5. Conclusions

CLIC1 expression was heterogeneous in intensity, distribution, pattern, and scoring in primary tumors and their corresponding liver metastasis with a digestive origin and a desmoplastic, replacement, or pushing pattern. The correlations between CLIC1 scores and the proliferative and non-proliferative microvascular density sustain the diagnostic, prognostic, and therapeutic importance of CLIC1 in liver metastases with different HGPs.

## Figures and Tables

**Table 1:** Distribution of mean values of microvascular density in primary tumors and their corresponding LMs, with a p-HGP.

Primary Tumor/Corresponding Pushing HGP of LM	CD34+/Ki67− Non-Proliferative Vessels; Average Values of MVD	CD34+/Ki67+ Proliferative Vessels; Average Values of MVD
Well-differentiated primary CRC/corresponding LM	4.33	0
	1.66	0
Moderately differentiated CRC/corresponding LM	16.8	3.2
	19.8	1.2
Poorly differentiated CRC/corresponding LM	27.25	2.5
	39	2.75
Moderately differentiated gastric adenocarcinoma/corresponding LM	62	12
	34	5
Poorly differentiated gastric adenocarcinoma/corresponding LM	32	7
	37	7

## Data Availability

Not applicable.
